# Enabling and inhibiting doctors transitions: introducing the social identity resource and belonginess model (SIRB)

**DOI:** 10.1007/s10459-024-10360-0

**Published:** 2024-07-24

**Authors:** Gillian M. Scanlan, Lisi Gordon, Kim Walker, Lindsey Pope

**Affiliations:** 1 Centre for Medical Education, University of Dundee, Dundee, UK; 2 Centre for Healthcare Education Research and Innovation, University of Aberdeen, Aberdeen, UK; 3 School of Health and Wellbeing, University of Glasgow, Glasgow, UK

**Keywords:** Transitions, Professional idenity, Wellbeing, Belongingness, Career intentions, Social support

## Abstract

The transition into postgraduate medical training is complex, requiring an integration into the workplace, adjustment to new identities, and understanding of the social and organisational structure of healthcare. Studies suggest that social resources, including a sense of belonging, inclusivity from social groups, and having strong social identities can facilitate positive transitions. However, little is known about the role these resources play in junior doctors’ transitions into the healthcare community. This study aimed to explore the implications of having access to social resources for junior doctors. This study undertook secondary analysis from a longitudinal qualitative study which followed 19 junior doctors (residents within two years of qualification) for nine months. Data were thematically analysed using an abductive approach, with the social identity resource and belongingness (SIRB) model as a conceptual lens to explore how social networks of support act as identity resources (IRs) for junior doctors as they experience transitions. The doctors narrated that having accessible IRs in the form of supportive workplace relationships enabled an integration and a sense of belonging into healthcare practice, supported the construction of new professional identities, and strengthened career intentions. Those with inaccessible IRs (i.e. poor workplace relationships) expressed a lack of belonging, and casted doubt on their identity as a doctor and their career intentions. Our study indicates that SIRB model would be beneficial for medical educators, supervisors, and managers to help them understand the importance and implications of having IRs within the workplace environment and the consequences of their accessibility for healthcare staff experiencing transitions.

## Introduction

Transitions are inherent across medical careers and can be simultaneously exhilarating and stressful (Brown et al., 2021; Gordon et al., 2017). Multiple, multidimensional transitions theory (MMT) defines transitions as continuous processes embedded within psychological, social, cultural, and educational adaptions that change over time depending upon context (Jindal-Snape, 2016). Transitions offer opportunities for personal and professional growth and the need to support transitions cannot be overemphasised. A new learning environment or job role could be a positive learning opportunity within a career pathway (Atherley et al., 2019, 2020; Coakley 2019). Evidence also shows that doctors are more susceptible to significant stress and burnout if transitions are inadequately managed at an individual and organisational level (Gordon et al., 2022; Lundin et al., 2017). The longer-term impact on staff wellbeing, retention and ultimately patient care is increasingly recognised (Monrouxe et al., 2017; Scanlan et al., 2019). Therefore, it is timely to understand how transitions are experienced in medical careers, what creates a sense of belonging for transitioning doctors, and how individuals can adapt to these workplace transitions. Drawing on social psychology theory, this paper utilises the Social Identity Model of Identity Change (SIMIC) to explore junior doctors’ social identity during early career transitions to identify factors that can promote successful transitions through training.

## Background

Junior doctors regularly rotate to new physical spaces (hospital, clinic, department, specialty), and need to adapt to each social and cultural environment. These transitions are often viewed negatively as frequent, short-term rotations can make integration into a workplace environment more challenging, and interpersonal relationships difficult to develop (Brown et al., 2021). Previous longitudinal research highlighted how interpersonal relationships in the latter stages of training can facilitate successful transitions (Gordon et al., 2017). Moreover, social integration processes can enable transitions through social support and relationships with those in the new workplace environment (Atherley et al., 2020; Yardley et al., 2018, 2020), all supporting a sense of belonging (Borrott et al., 2016; Ching et al., 2022; Waller, 2020). Sense of belonging (SOB) is the feeling, internal belief, and expectation that a person fits and is included and connected to a group or community (Levett-Jones & Lathlean, 2009; McMillan & Chavis, 1986). A feeling of personal involvement and shared or aligned personal and professional values with a group helps individuals to identify as an integral and valued part of that community (Hagerty et al., 1992; Levett-Jones & Lathlean, 2009). It can better support interpersonal relationships, academic success, career achievement, and health and wellbeing (Bentley et al., 2019; Hagerty & Patusky, 1995; Hagerty et al., 1992; Jetten et al., 2015; Ma, 2003). Our conceptual framework, described next, explores the relationship between an individual’s social identity and their sense of belonging as they experience transitions.

### Conceptual framework: social identity model of identity change (SIMIC)

SIMIC, originating from Social Identity Theory (SIT) (Tajfel & Turner, 1979), outlines how a person’s identity can be empowered by others and the groups in which they belong. It recognises that a person’s identity is continually constructed and reconstructed throughout daily life, based in the context in which they find themselves (Turner et al., 1987, 1994). For example, a parent dropping their child at nursery would likely define themselves differently in this context compared to entering the medical ward ready to begin their shift as a doctor. These changing self-definitions have a direct impact on how people think, feel, and behave (Turner, 1982; Turner et al., 1994). SIMIC recognises personal and social processes, how identities are influenced and determined by personal traits and characteristics, and the social groups and memberships to which individuals belong (Cruwys et al., 2020). It unpacks why group affiliation is important throughout life, considering when and in which contexts these groups influence individuals’ identities (Haslam et al., 2021). SIMIC highlights how social groups can support positive outcomes when individuals are experiencing major life and identity transitions (Praharso et al., 2017; Bentley et al., 2019). These supportive social networks and processes are referred to as identity resources (IRs). Individuals who have access to multiple identity resources (IRs) are better able to navigate significant life transitions, new identities and roles. For example, group connections can influence individuals across several domains in their life, including appraisal of physical and psychological health, how they navigate educational, career and family transitions (Bentley et al., 2019; Cruwys et al., 2020; Iyer et al., 2009; Praharso et al., 2017). Researchers studying transitions into university found that when undergraduate students developed new social groups and were able to maintain their pre-existing ones (i.e. multiple IRs), they were more likely to adapt to transitions successfully and have better health outcomes (i.e. lower depressive symptomology) than those who are not able to do so (Iyer et al., 2009; Cruwys et al., 2020, Praharso et al., 2017). Identity construction also relies on a person’s perception of how well they will be able navigate a compatible pathway between their current and future self (i.e. trainee doctor to consultant) (Bentley et al., 2019). The wider educational literature suggests research students who were able to draw on their multiple identities (i.e. student, scholar, researcher) coped better with uncertainty and were more likely to perceive a compatible career pathway both now and for their future self (Bentley et al., 2019). Those lacking a strong personal identity, and expressing higher levels of doubt in their capabilities, had difficulties developing group affiliations and perceived their own identities to be incompatible with leaders in the profession (Bentley et al., 2019). This research suggests that the multiple identities people access throughout their career may play an integral role in their ongoing professional identity development and career intentions. SIMIC supports this; successful transitions will be facilitated when individuals can access multiple social groups and social identities (i.e. multiple IRs), both pre-existing and new, playing a critical role in facilitating new personal and professional identities, integration into a community and ultimately a sense of belonging (Iyer et al., 2009; Jetten et al., 2015; Praharso et al., 2017). Underpinned by SIMIC, our study aimed to explore junior doctors’ experiences of early transitions into the medical community. We asked: In this context, what are IRs and why are they important to junior doctors throughout this time; in what ways do IRs enable or inhibit these transitions into the medical community; and what are the consequences of IRs for junior doctors during these initial transitions into the medical community? We analysed data from a longitudinal qualitative study following 19 junior doctors (residents within two years of qualification) for nine months. In this paper, using our data, we articulate key IRs junior doctors need and the implications these IRs have on individuals undertaking initial transitions into the medical profession. Following our analysis, we present a new conceptual model (the Social Identity Change and Belongingness Model) (SIRB) which will aid medical educators’ understanding and provide insight into supporting doctors’ transitions.

## Methods

### Context

The data for this study is drawn from research to develop evidence-based interventions to support doctors’ wellbeing during transitions (Gibson-Smith et al., 2022; Gordon et al., 2022). In the UK (the context of this research), following graduation from medical school, doctors work in the Foundation Programme (FP) for two years full-time equivalent. This provides broad-based training with doctors rotating across a breadth of specialties in typically four month placements, aiming to develop the necessary professional skills and competencies prior to entering a specialty training programme. As this study took place during the COVID-19 pandemic, there was some disruption to the delivery of the FP including changes to teaching and learning opportunities or scheduled rotations (e.g. doctors spent longer in a specific placement than expected or were relocated to a different specialty).

### Design

This research is epistemologically grounded in social constructionism (Burr & Dick, 2017), recognising meaning as constructed through social interaction. Multiple and muti-dimensional transitions theory (MMT, Jindal-Snape, 2016) informed the study design, influencing the questions we asked participants and how we articulated transitions to them. Following ethical and institutional approvals (as part of the larger study), potential participants were invited by local NHS boards and educational bodies via an email disseminated to doctors across Scotland through several channels (e.g. training programme directors, clinical and educational supervisors, foundation programme directors etc.). Additionally, a social media call invited Scottish doctors to participate in a longitudinal audio diary research study. A total of 19 FP doctors agreed to take part in the four-phase study exploring their transitions during the COVID-19 pandemic, this included: (1) an entrance interview; (2) a 6–9-month longitudinal audio-diary (LAD) phase; (3) a second interview and (4) a third interview. Interviews and LADs allowed in-depth exploration of lived experiences through time, facilitating participants sharing in-the-moment reflections of their story to researchers as well as changes through time (Monrouxe, 2009, Crozier & Cassel 2016; Williamson et al., 2015).

### Data collection

For this paper, in-depth secondary data analysis was undertaken for FP doctors involved in the primary study between June 2020–March 2021. 19 foundation doctors were involved, (see Table 1 for a breakdown of participant characteristics). For this paper, a total of 2900 min and 30 s of audio-data were analysed. There were 19 first interviews (average length 53 min); 13 s interviews (average length 1 h 5 min); 12 third interviews (the average length 1 h) and 14 participants submitted at least one audio diary, totalling 45 audio diaries.

**Table 1:** Junior doctors characteristics

Gender	
Female	14
Male	5
Age	
Under 25	12
26–34	7
Nationality	
United Kingdom	12
Irish	2
European	2
South-East Asia	2
Missing	1
Ethnicity	
White	16
Asian/Asian British	2
Mixed/Multiple ethnic	1

### Specific ethical considerations

This study was approved by the University of Aberdeen College Ethics Review Board (CERB/2020/5/1985) and received NHS R&D approval (IRAS ID 284599). Within this, consent was given by participants of the larger study that their data could be used for secondary analysis. As part of standard protocol, the research team provided a distress pathway in the participant information sheet advising participants how and where they could seek support if required.

### Data analysis and development of a novel conceptual model

**Fig. 1:**
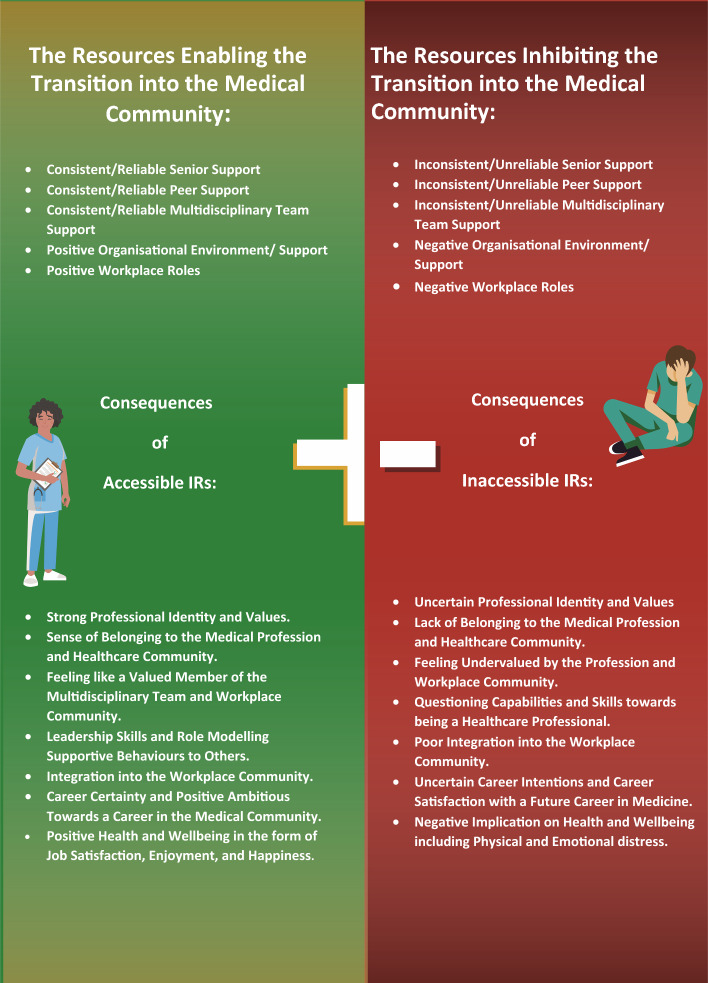
Social identity resource and belonginess model (SIRB)

Initially framework analysis was used across the larger data set to abductively identify broad themes. This analysis involved a number of stages, including: members of the wider research team familiarising themselves with the dataset through reading through extracts of the data and via weekly team discussions; a thematic framework was developed to code a sub section of the data using NVivo 12 qualitative data analysis software (Lumivero, 2017); coding was then confirmed in discussions during data analysis sessions, where code meanings and data examples of these codes were discussed; through repeated (weekly) discussions with the data collection team, more analysis was undertaken on the large data set that focussed on higher order themes. The overarching themes focussed on the transitions that doctors (across all career grades) had experienced throughout the pandemic and the implications these transitions had on all areas of their lives (Gordon et al., 2022). A key primary theme in the initial data analysis was around ‘supporting transitions’. The authors of this paper undertook further in-depth secondary analysis of the key theme ‘supporting transitions’ focusing on junior doctors using SIMIC as our conceptual framework. On scrutinising the data, we discovered transitions experienced by junior doctors were often directly influenced by the social and organisational relationships/networks of support they developed throughout the initial stages of postgraduate training (i.e. the MDT, fellow junior doctors, clinical/educational supervisors, NHS Board, training programme, the hospital etc.). Further exploration indicated these relationships/networks of support would either enable and support these transitions or could negatively inhibit these. We recognised many of the key components of the SIMIC model across our data set. However, we also noted that sense of inclusion and belongingness played a critical role in successful transitions into the medical community. We reflected that the healthcare context relies upon workplace social interactions in a team-based environment, something that is a vital factor in the transitions experienced throughout healthcare careers. Thus, for the purpose of application to the transitions experienced in the healthcare context, we extended the current SIMIC model to the ‘social identity resource and belonginess’ model (SIRB), depicted in Fig. 1. We drew on this novel conceptual model to help us to understand and explain the data through cross sectional and longitudinal analysis to explore the implications of these networks of support.

### Reflexivity

The all-female research team were experienced healthcare researchers from 3 Scottish medical schools. Two (LP, LG) have a clinical background, one (GS) has a psychology background and one (KW) a pharmacology background. Our multidisciplinary research team, consisted of 4 white women with expertise in the application of social constructionist approaches and published work on junior doctors’ experiences in postgraduate training; transitions, general practice; evidence and theory-based intervention development; qualitative interview techniques and audio diaries methodological approaches. Whilst acknowledging our research team diversity in research experience and appropriate expertise, our personal and professional backgrounds will have inevitably influenced how we conducted our analysis. For example, it is important to acknowledge that as an all-white research team our interpretations will come from this perspective. Inherent in our research approach, we also acknowledge that we hold power through our role and the choices we made in study design, the questions that we asked, our interpretations of the data, and how and what we choose to present in our findings, including the pseudonyms we gave participants. To help understand our roles in the research process and to encourage supportive team reflexivity, we met regularly to discuss data analysis, our own experiences and understandings of transitions, and the application to the study context (Olmos-Vega et al., 2022).

### Findings

We begin by outlining SIRB as applied to junior doctors before describing multiple participants’ experiences to illustrate how IRs enable and inhibit transitions into the medical community and the resultant consequences of access to these resources (see Table 2). To further illustrate the complexity of individual experiences of transitions longitudinally, we present one participant’s experience (see ‘Sara Story’, Table 3). Her story shared commonalities with other participant experiences and provides multiple examples of how social and organisational networks impacted on her transition into the medical profession. Exploring Sara’s longitudinal experience in-depth enhanced understanding of the importance that junior doctors place on IRs and the ways in which they can support successful transitions. In this paper we use pseudonyms to bring the raw data to life capturing the richness, depth of participants’ lived experiences and social worlds; we deemed the use of participant identifiers (i.e. participant one) be too impersonal and lacking in authenticity (Heaton, 2022). To help depict a comprehensive understanding of the data, we present the conceptual model of SIRB as applied to our data, alongside extracts from the dataset reflecting the different aspects of the model.

**Table 2:** Additional examples of SIRB applied across the data set

Juan—F2—interview 2	Quote 1	Two of them were relatively new nurses, Once he died, she was quite upset, and I think from my experience, from people coming over to me and saying do you need a minute, do you need a hand, I throughout the night asked her if she needed help with her jobs, asked her if she needed a minute… afterwards… I said to her, well done, you did very well…there was nothing we could have done
		**Key components of SIRB**—**IR ‘Workplace Role’ enables the transition**—**consequences -development of leadership and professional identities within the workplace, integration into the medical community and offering informal support to colleagues.**
Graeme—F1—third interview	Quote 2	In my previous job I had a fantastic time with all the other colleagues that I worked with. I was well loved by the nurses. So, I don’t know what has gone wrong… I feel that I was quite surprised at how much my difficult interactions with my colleagues in my current work have impacted my wellbeing. In my previous job…coming to work actually boosted my happiness because I loved my job so much…
		**Key components of SIRB**—**IR ‘MDT’ inhibiting the transition**—**consequences**—**reduction in job enjoyment/satisfaction, implications on psychologically wellbeing.**
Angela—F1—third interview	Quote 3	I got a phone call basically from the actual clinical director basically saying, look, we don’t have a specialty doctor anymore…I really need some help from a physical health perspective, and you are it basically…It was lot of responsibility because everyone basically was looking to me to make clinical decisions… And I had a lot of support in terms of like senior support, emotionally and trying to help me…the charge nurses were amazing, like as soon as they knew what they had to do they did everything… They were amazing… But the team were great…
		**Key components of SIRB**—**accessible IRs (Senior team, MDT)**—**consequences**—**a sense of value, integration into the clinical environment, job enjoyment and strengthening of professional identity and leadership skills.**
Elizabeth—F2—audio diary 1	Quote 4	’I’m working in the acute receiving unit…I felt very stressed and very unenthusiastic about medicine and I also think I felt quite undervalued…I had hoped to sort of explore my future career and my future within medicine this year and I am kind of at the moment wondering if I have a future in medicine…I’m kind of questioning myself as a doctor and how well I’m managing the transition here and I just don’t feel particularly confident in myself or my decisions
		**Key components of SIRB—Inaccessible IRs (‘organisational support’)—consequences **—**lack of belonging and sense of value, uncertain career intentions and questioning professional identity/capabilities of being a doctor.**

**Table 3:** Sara’s longitudinal journey

Month of study	Data excerpt	Longitudinal interpretation	SIRB model in action
Month 1: initial interview	*I’ve got really lovely FY1 and FY2 colleagues who, I think, through working up through it all, we’ve all become quite close… we’ve all worked quite cohesively together to try and drive positive changes for the department… a sort of quality improvement project, we were able to split our teams and make things better. I took on too much, basically, so other people stepped in. Someone’s doing the rota now, so it’s all split quite well, and my colleagues have been really personally supportive as well. When I was off sick, they were like, “Just take your time,” you know, because I felt awful leaving them short, but they were so kind about it. So I think that has really been the saving grace of it, to be honest, just working with some really lovely people*	In her initial interview, Sara spoke candidly about the importance of peer relationships in her workplace. She reflected these relationships were a ‘saving grace’, highlighting them acting as IRs in her early transitions into the medical community.	The IR of ‘Peer Relationships’ enabling the transition into the healthcare community. Consequence—feeling valued and supported by the organisation and developing a sense of belonging.
Month 1: audio diary 1	*What surprised me quite a lot is I was actually off for about three weeks about a month and a half ago with really severe anxiety… a lot of the fallout from the change, the burden from that was being placed on the FYs shoulders and we were expected to sort it out. Very little in the way of senior presence both physically on the wards, but also just from that kind of more managerial level…When I came back to work, no-one checked how I was getting on, how I was performing and actually…it would just be nice for someone to check in and ask if I was okay…we don’t feel as valued by our seniors or by the management as we would like to be. Not for the job that we get paid to do, but just for being human beings*	In Sara’s first diary she shared the ‘disappointment’ experienced when she returned to work after a period of sickness, as no members of management checked to see how she was coping on her transition back to the workplace. In line with SIRB, it is clear from this experience that a lack of IRs enables a sense of exclusion from the workplace community. Sara comments on the importance of feeling like valued ‘human beings’ perhaps highlighting the psychological and physical toll of not having accessible supportive IRs during these transitions.	Inaccessible IRs in the form of senior and organisational support inhibiting the transition. Consequence—sense of exclusion from the workplace, physical and psychological toll.
Month 2: audio diary 3	*I think a really positive thing that’s come out from it is that our relationships amongst the junior levels specifically the junior levels, so with fellow FY1’s and FY2’s and the reg’s, they’ve been much improved. I think there’s that sense of solidarity. People have been really looking out for each other. People have just been so kind and supportive. I think part of that is because we’ve been spending a lot more time together…I think there’s been that recognition that, yeah, we’re all human and we are all struggling in our own ways, both professionally and personally*	In month two Sara highlighted the importance of the continuity of workplace peer relationships. These relationships are not only important to develop her professional role as a doctor and develop a sense of belonging to the medical team, but they also serve as a social and psychological support base in her personal life. This fits with the important components of SIRB around the importance of maintenance of both new and old relationships as individuals experience transitions. The continuity of new relationships has a positive implication, not only on the experiences of the junior transitions, but on Sara’s health and wellbeing.	Accessible IRs in the form of peer support enabling the transition. Consequences—integration into the workplace team, strengthened PI and supporting health and wellbeing.
*End of month 2: audio diary 5*	*I’m a little bit scared of work now, given… all the multiple issues I experienced …it’s starting to feel a bit less like a vocation and more like a necessity, which is disappointing and I really want to find that spark again…I love the job itself. I love what it entails. I love looking after people. I love that side of things and the compassion, but what I hate is the bureaucracy of it all… Sometimes I just feel like a little bit of a shadow of a kind of the bright, bubbly person I was…. A big part of the reason I went in to medicine is I wanted a job that made me feel like I was adding something, you know a positive contribution to the bigger picture of life I guess, like you know I wanted to feel like I was doing something worthwhile for, you know on a deeper level, in my purpose and I just, I suppose you don’t feel valued a lot of the time… if people aren’t valued in their job and if they don’t have autonomy….and I do feel like sometimes if I’m like not at work, I’m like oh god I’m a waste of space and I’m not doing my duty in a way…I feel like I’ve lost a lot of passion for the job… because I’ve lost faith in the system…I don’t want to have to give so much of my own life, you know you make so many sacrifices for a service where I essentially felt quite, well you are essentially dispensable*	Towards the end of month two, coming to the end of her first two years of postgraduate training, Sara indicated that she has ‘lost faith in the system’. Based on Sara’s articulation, being a doctor is part of her professional and personal identity, however, the lack of compatibility between these two roles causes internal conflict. This is particularly evident when she questioned her duty as a doctor in her free time and described feeling like ‘a waste of space’. Viewed through the lens of SIRB, Sara’s ‘new’ professional identity of what it means to be a doctor is incompatible with her own personal beliefs of what she expects the role of a doctor to be. The perceptions of incompatibility may also stem from not feeling closely aligned to the ‘typical’ members of the profession, attributes associated with these individuals and the expected workplace role of a doctor. This appears to reduce a sense that this career path is feasible and undermines her motivation to pursue this pathway.	IR in the form of ‘workplace role’ inhibiting the transition. Consequences—psychological toll, a lack of job satisfaction and enjoyment, feeling undervalued by the profession, questioning PI.
Month 4: second interview	*Oh, I just loved it… getting to enjoy my work again…the other thing I noticed was like the second I walked into the ward I was like ‘this is a much happier working environment’…And it kind of made me realise, no this is actually what I want to do, this is what I love doing. But you don’t realise that when you’ve been stuck in somewhere for eight months that’s quite dysfunctional….I think it’s just things like having a consultant or someone more senior than you that’s actually interested in you and how you’re getting on. Like, a genuine interest… Not only in their education but also in their, kind of, wellbeing…And another good thing… the doctors’ mess, was every doctor from every level was on it…like you learned so much wisdom aside from your knowledge. But see just about being a doctor, speaking to consultants and registrars. Just like, casually…You got some much life advice and you know, even talking about things like what’s it like to have children as a doctor… You know, the life outside work*	Between month four and five, Sara started a locum post in a large district hospital. In the second interview she expressed that this new post made her realise that she wanted to be a doctor again. She remarked about the positive working environment, including peers and senior members offering personal advice about the role of being a doctor. This fits with SIRB, in that having multiple IRs allows juniors to thrive during transitions. This could be through further development of group affiliation, the ability to observe and interact with members of the healthcare team, and the opportunity to engage with role models that allow juniors to construct their own perceptions and aspirations of their professional identity now and in the future.	IRs in the form of ‘organisational support’, ‘senior support’, ‘peer support’ supporting the transitions. Consequences—career certainty, job satisfaction, improved health and wellbeing, strong PI.
Month 9: third interview	*… I really enjoy my clinical work a lot more. I get a lot out of it and I'm like, this is what I want to do. I love it. I really enjoy being a doctor but it’s made me realise that maybe having a… job where I can have some time to do teaching and not a hundred per cent clinical all the time might be the way to go. And I think…going off the training pathway for a bit has made me realise that you can do what you want… also taking the time for myself makes me perform 100% better than I would when I’m burnt out and I just enjoy everything better. And I think…there’s no one path through medicine. You can do lots of things. You can form your own path…I was hoping that maybe I could become a speciality doctor in palliative medicine which is what I really enjoy… …we’ve been sold this line that you just need to keep going no matter what. And…it’s not true. Like, you can do it if you want but you probably won’t be very happy or fulfilled…So I don’t care if my GP training takes me ten years to get though if I end up having children or whatever, I literally don’t care how long it takes me to get there. It’s good to be accomplished and at the top of your career ladder at some point but I have no goal to get there imminently or as quickly as I can. I’ll just take my time getting there*	In month nine, during the final interview with Sara, it appeared that her new clinical development fellowship post and the previous locum experiences re-ignited Sara’s passion for being a doctor, her future career plans, and intentions.	Multiple IRs in the form of ‘organisational/workplace environment’, ‘workplace role’, supporting the transition. Consequences—career certainty, strong and confident PI, job enjoyment and satisfaction.

### Summary of the SIRB applied to postgraduate training transitions

Junior doctors described multiple IRs and their impact on transitions. In this context, IRs refer to the support networks accessible within their working and learning environment. These networks of support included, but were not limited to, the healthcare team (e.g. nurses), peer support (e.g. fellow junior doctors), senior support (e.g. clinical supervisors) and organisational support/environment (e.g. the NHS board/ training programme/hospital/clinic). In line with SIMIC, these networks of support become IRs when an individual is going through significant work-life transitions, such as the progression to the next stage of training or a new placement. Our data set evidenced that these IRs enabled or inhibited the development and facilitation of their identity as a ‘doctor’. In reference to Fig. 1, the SIRB model shows how IRs can enable positive transitions into the medical community with implications across several domains (i.e. sense of belonging to the medical profession, identity development etc.). Figure 1 also shows that IRs can inhibit transitions with negative implications on a personal level (i.e. uncertainty around career intentions, professional identity struggles, challenges to wellbeing).

### Illustrative data examples

Here we present examples from the dataset illustrating the SIRB model, which included: specific IRs that junior doctors accessed; how these IRs enabled or inhibited transitions into the healthcare community; and the consequences associated with these IRs. The IRs described are exemplified throughout this findings section (i.e. in the data illustrations and in the longitudinal stories shared). Table 2 adds examples of SIRB from across the data set.

#### Example of senior support as an IR:

Educational and clinical supervisors acted as IRs, playing an integral role in creating a sense of belonging and value within the workplace. Celina articulates how her supervisory relationship made her transition into postgraduate training easier:

… one of my supervisors, she made it a point to remain as…one of my supervisors… I thought that was really great. There’s no reason for her to have…but she insisted that she stay, and I think that aspect of consistency did help… She’s also checked in on me…I think that has been quite exceptional and quite helpful.

***(Celina, F1, Initial Interview – IR in the form of Senior Support—consistency and accessibility of this IR enables the transition)***

#### Example of peer support as an IR:

The continuity and maintenance of relationships over longer periods of time helped juniors to establish collaborative and supportive working. Gerard provides an insight into the importance of peer relationships:

the opportunity to sit down and chat to some people…A lot of people are just interested to find out how things are going for each other…as a junior doctor you just get four months somewhere and it’s not really that long to make a relationship, whereas now that I’ve been here for eight months, I’ve made stronger friendships and relationships with some of the nurses than I would have done…

***(Gerard, F2 Initial Interview—IR in the form of Peer support—continuity and accessibility of this IR enables the transition into the healthcare community)***

The continuity of these supportive relationships could help juniors understand the differing roles and responsibilities expected of them both now and in the future.

#### Example of the MDT enabling the transition into postgraduate training:

Most junior doctors described how feeling part of the multidisciplinary team (MDT) enriched their training experiences, initiated integration into the workplace community, and facilitated sense of belonging to the medical profession. Simon articulates the importance of the medical team in shaping his perspective on what it means to be part of the medical profession:

I like being part of the team…I like the diversity within it and the fact that you… have from the consultant to the… least senior member of the team. … by the end of it…I felt part of the team and I felt like I was a valued part of the team. And I felt like I did do a good job, …the way…the nurses – because…they respected, like, the way …I did my job, …I got really good feedback from all of them.

(**Simon, F1, Third Interview—IR in the form of the MDT—the accessibility of the MDT enables an integration into the medical community—consequence—feeling valued and appreciated member of the healthcare team**).

The team made a conscious effort to integrate him and he described ‘value’ and ‘respect’ as a key contributor to feeling part of the community. The team structure and the people within it acted as IRs creating a sense of belonging and inclusion and enabling construction of new identities (i.e. what it means to be a junior doctor). A positive network of support from the workplace community appears to instil a sense of value in the individual role as ‘doctor’ but also makes visible the importance of the job they undertake, helping early career doctors to cope even in the most unfamiliar or challenging situations (see quotes from Juan [Table 2, quote 1] and Angela [Table 2, quote 4]). From the perspective of SIRB, the relationship with the MDT enables a sense of support and integration, and facilitates the development of new identities in the workplace.

#### Example of positive consequences of having accessible IRs available:

Doctors able to access IRs in their working and learning environment, while afforded the opportunity to work autonomously, felt more confident in their role as doctors and more satisfied with their job role. Graeme explains:

I never had this level of independence in my previous job…I really enjoy being able to work independently and basically feel very much like a proper doctor, being able to independently diagnose things that are going on and trying to figure out what’s going on…I get to meet an entire different bunch of colleagues who have been very supportive.

***(Graeme, F1, Third Interview, Accessible IR in the form of ‘workplace role’—consequence—strong and confident PI and Job fulfilment/enjoyment).***

Similarly, Angela (Table 2, quote 4) narrates her experience of increased clinical responsibility enabled by the supportive team around her. Having accessible IRs helped the doctors construct their new identity and understand the key attributes needed to be a ‘proper doctor’, easing their transition into postgraduate training and integration process within the medical community.

#### Example of IRs inhibiting the transition and the negative consequences of inaccessible IRs:

Junior doctors without accessible and supportive IRs in the form of a supportive team environment found integration into the workplace community challenging. Some described feeling a lack of belonging and that their role within the medical community was undervalued by the wider health care team and organisation. At times, the apparent disconnect between expectation and reality for junior doctors was challenging. Alongside a lack of access to peer support and/or supportive senior staff in their immediate surroundings, the new demands and expectations of their junior doctor role could become overwhelming. Ling highlights the negative implications of a lack of a supportive working culture on her ability to cope physically and psychological with her new doctor role:

**Ling**: We don’t really have F2s and other juniors within the ward over here… So I will be the only one…I am struggling a little bit… it was a lot… just really hoping that I’m doing okay… It’s a bit tiring… feeling that I’m constantly pushing myself beyond my limits… And sometimes at work, it’s…in terms of receiving feedback…a lot of feedback most of the time…or feedback of things that…in areas that I could improve in, it feels a bit as though…oh my, sorry (Ling cries)… I am filling up a little bit (Ling cries), I’m not quite sure why… It does feel that… day after day I’ve been trying (Ling Cries)…

Speaker: Take your time.

Ling: I’ve just been trying really hard and doing my best and it does feel sometimes that it’s not enough…I seem a bit stressed and overwhelmed, I can’t…I just…I can’t (Ling cries)

(**Ling, F1, third Interview—Inaccessible IRs in the form of ‘peer support’, ‘senior support’ and ‘organisational support’ inhibits the transitions into medical community—consequence—’identity’ struggles and negative implications on health and wellbeing**).

Ling questions her ability as a doctor on multiple occasions. The lack of positive affirmations from senior staff around her performance may have exacerbated this. With little or no access to IRs due to poor interpersonal relationships and/or not feeling a sense of support from the workplace team (i.e. nurses) and/or organisation (i.e. the hospital where they work, the foundation training programme in which they are part of), junior doctors can question whether they fit within the healthcare team and their identity as a doctor, also the case with Elizabeth (Table 2, quote 5).

Ling and Elizabeths’ narratives provide insight into how negative interactions and disconnected relationships can have negative implications on job enjoyment, taking a psychological and professional toll. There is a sense reciprocal relationships within the workplace are linked to job satisfaction and the wellbeing of employees, for example as narrated by Graeme (Table 2, Quote 3). These examples highlight the role of developing and maintaining new social groups when individuals are going through work-life transitions (i.e. medical student to junior) and the negative implications on transitions if these IRs are not available.

### Sara’s story

We now present Sara’s longitudinal story which highlights the consequences associated with accessibility of IRs during transitions. Sara participated in this study for 9 months, undertaking an initial interview, 6 audio-diaries, a second and third interview. Sara was in the final rotation of her FP training when she was first interviewed. During month two of the study, she moved to a new city to be closer to family and friends with her fiancé. During months 4–6 she worked as a locum doctor in a medical ward in a large district hospital and during months 6–9 worked as a clinical development fellow in medicine (see Table 3 for Sara’s story including illustrative examples of data across multiple timepoints).

## Discussion

To the best of our knowledge, this is the first study to explore what IRs are for junior doctors and how the consequences of IRs impact on transitioning identities in early medical careers. In the context of this research, IRs referred to the networks of support that foundation junior doctors had access to within their working and learning environment. These IRs either enabled or inhibited the development and facilitation of their identities as doctors. This study indicated that accessible IRs enriched training experiences, initiated integration into the healthcare community, enabled a sense of belonging and inclusion within the workplace environment and facilitated the construction of new professional identities. For instance, if multiple group affiliation and belonging can help facilitate a sense of continuity between past and present, it may also be able to act as a bridge to the future (Haslam et al., 2021). Individuals who were able to access multiple identities (e.g. junior doctor, team member, mentor, peer) in the context of their working lives, both in terms of the number of groups to which they belong and the number of ways they understand who they are and what they do, may also be able to construct future identities (e.g. consultant, general practitoner) (Bentley et al., 2019; Cruwys et al., 2020). Exploring the data both cross sectionally and longitudinally offered in-depth and nuanced exploration of early career doctors’ transitions into the medical community. Our data indicated that inadequate IRs can be detrimental. Junior doctors lacking adequate IRs may struggle to identify their role within the workplace environment and grapple with where they fit within the organisation, and ultimately the profession. This made it challenging to construct their professional identity both now and in the future. Without adequate IRs, junior doctors felt isolated and lacked a sense of belonging to the medical community. This could have knock-on consequences on their wellbeing and future career intentions. The social identity literature enables us to understand and unpack the complex nature of life transitions, recognising that when individuals are undergoing life transitions, they also experience identity transitions (Haslam et al., 2021). The SIRB model acts as a useful guide for medical educators, supervisors, and managers to consider IRs and the consequences of accessibility or inaccessibility and how this might affect staff experiencing work-life transitions. Whilst this study focussed on junior doctors, we see the potential applicability of SIRB to doctors across the career continuum and other healthcare professions. Our study demonstrates the importance and reliance of workplace social interactions in team-based environments, something even more critical for learners experiencing transitions across their healthcare. Our study shows how social processes were a vital component to enable or inhibit transitions. Our dataset was diverse in terms of gender and background characteristics of junior doctors. However, we acknowledge certain limitations in transferability due to variability in healthcare contexts and training programmes. Our work was undertaken in a Western context which may limit transferability to non-Westernised contexts. This is particularly relevant within the context of wellbeing where there can still be stigma associated with accessing and accepting support. It was out with the scope of our study to explore differences in experiences between those from differing cultural backgrounds (e.g. race, ethnicity, socio-economic status). Whilst every individual’s experience is unique, further research is needed to explore how cultural background might influence accessibility to IRs as well as the application of SIRB across international contexts, the career continuum, and with other healthcare professionals. We acknowledge the potential influences of our professional and personal backgrounds and life experiences on our data interpretations and recognise that our theoretical choices and conceptual framework led to a specific focus on personal and social aspects of transitions. Exploring the data through another lens (for example socio-materiality or cultural aspects) may have added additional layers to our analysis and may be a direction for future development of the SIRB model. We also acknowledge the power relationships inherent in this type of research through the choices we made throughout out research. For example, the research team chose participants’ pseudonyms, which is an expression of our data interpretations (Allen & Wiles, 2015). As a team we maintained a reflexive approach meeting regularly to discuss our work before during and after this research (Olmas-Vega et al. 2022). We have also presented our work in many fora (e.g. to junior and senior medical staff, healthcare managers, and policy makers), using these opportunities to ‘sense-check’ our findings and allowing them to influence our conclusions, these processes help reduce this power differential. Our findings have policy, practice, and educational impact for health professions education. We can infer from our findings that strong interpersonal relationships with colleagues can positively impact an individual’s sense of belonging and value within the workplace environment. This reinforces the critical importance of consciously supporting newcomers in their transitions into the healthcare system (Monrouxe et al., 2018), particularly as early encounters within healthcare practice can have implications on professional identity (Brown et al., 2021) and career intentions of junior doctors (Scanlan et al., 2018). Moreover, our study indicates that SIRB could be used by medical educators, supervisors and those working directly with junior doctors (I.e., MDT) as a framework to help them understand the importance and implications of IRs within the workplace environment. This study adds comprehensive understanding of the imperative role of social processes play in enabling or inhibiting transitions in the workplace environment. Our findings indicate the need for compatibility between social processes and professional identities. Lack of compatibility leads to internal conflict, impacting whether an individual perceives their identity to befitting within the healthcare community, their self-concept of what it means to be a doctor, and whether a career in healthcare aligns with their personal and professional values and traits. Given the exodus of staff leaving the healthcare system (Poon et al., 2022), it is critical, policy makers, managers, and medical educators have a more holistic understanding of the role social dynamics play in identity construction and the interplay between the individual, the people, and the workplace environment. The SIRB model serves to highlight the social and environmental factors that influence career intentions and may facilitate retention of a highly skilled workforce.
